# Supplementary material for: Chaperone dependency during biogenesis does not correlate with chaperone dependency during refolding
Source: Mol Syst Biol. 2025 Oct 28;22(1):139–64. doi: 10.1038/s44320-025-00166-6 (PMC12759075; doi:10.1038/s44320-025-00166-6)
Supplement: Supplementary file 1 — Appendix [file 44320_2025_166_MOESM1_ESM.pdf]

Appendix for:

Chaperone Dependency during Biogenesis Does Not Correlate with Chaperone Dependency during Refolding

Divya Yadav[1], İdil I. Demiralp[1], Mark Fakler[1], Stephen D. Fried[1,2]

1. Department of Chemistry, Johns Hopkins University, Baltimore, MD 21218, USA
2. T. C. Jenkins Department of Biophysics, Johns Hopkins University, Baltimore, MD 21218, USA

Table of Contents

Appendix Figure S1. Temperature-dependent LiP-MS changes in WT. . .2

Appendix Figure S2. Scarless genomic tagging of *pgk*. 3

Appendix Figure S3. Biochemical and biophysical validation of tagged PGK. .4

Appendix Figure S4. Functional validation of *in vitro* translated PGK. . .5

Appendix Table S1. Primer sequences used in this study. 6

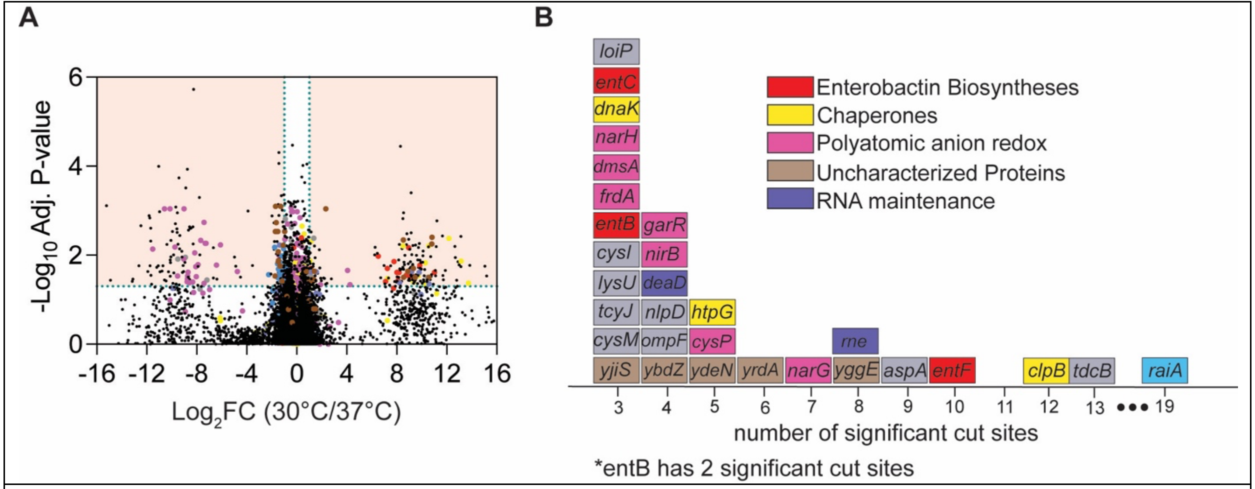

Appendix Figure S1. Temperature-dependent LiP-MS changes in WT. (A) Volcano plot of LiP-MS peptide features from wild-type *E. coli* grown at 30 °C versus 37 °C. The x-axis shows log2 fold-change (30 °C/37 °C) and the y-axis shows –log10 adjusted *P* value. Points are colored by the functional category of the parent protein (key at right). Peptides with >2-fold change and $p < 0.05$ (Welch's *t*-test following protein-wise Benjamini-Hochberg FDR correction) are highlighted in pink-shaded regions. (B) Proteins with significant LiP-MS perturbations between 30 °C and 37 °C, ranked by the number of significant cut sites (≥ 3). Bars are colored by functional category to match panel A (Enterobactin biosynthesis; Chaperones; Polyatomic-anion redox/transport; Uncharacterized proteins; RNA maintenance). Labeled tiles denote gene names. *entB* has two significant cut sites and is noted for completeness. See Source Data for Figure S1.

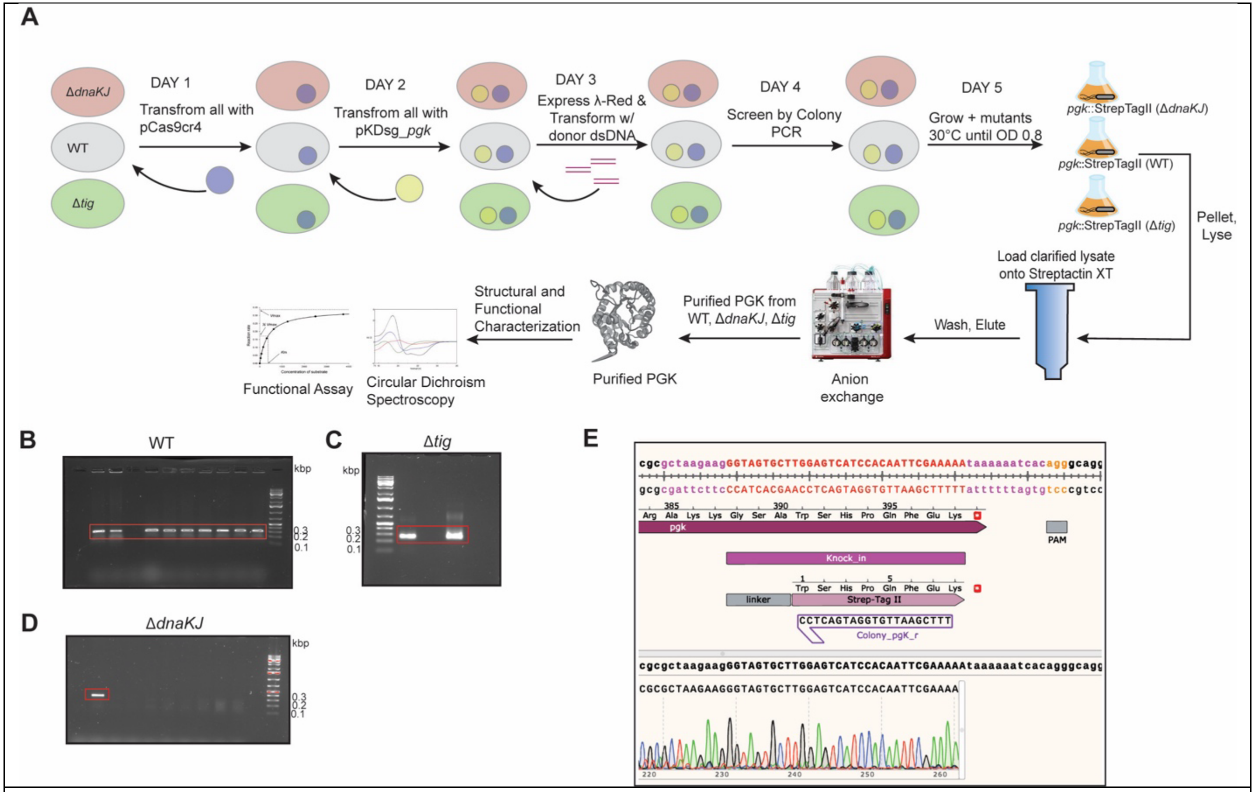

Appendix Figure S2. Scarless genomic tagging of *pgk* in WT and chaperone knockout *E. coli* strains for downstream structural and functional characterization. (A) Schematic overview of the CRISPR-Cas9–assisted, scarless genomic tagging workflow used to insert a C-terminal Strep-tag II at the endogenous *pgk* locus in WT, Δ*dnaKJ*, and Δ*tig E. coli* strains. The strategy involves sequential transformations using a λ-Red recombination system and a two-plasmid setup—pCas9cr4 for Cas9 expression and pKDsg_*pgk* for sgRNA delivery along with donor double-stranded DNA (dsDNA). Mutant clones were screened via colony PCR, and the tagged PGK protein was expressed and purified from clarified lysates using StrepTactin XT affinity chromatography followed by anion exchange. (B–D) Representative colony PCR gels showing successful integration of the tag at the *pgk* locus in WT (B), Δ*tig* (C), and Δ*dnaKJ* (D) strains. (E) Sequence confirmation of correct C-terminal tagging of *pgk* using Sanger sequencing. The integration site, coding sequence, and inserted Strep-tag II are annotated, with the PAM site and linker sequence highlighted.

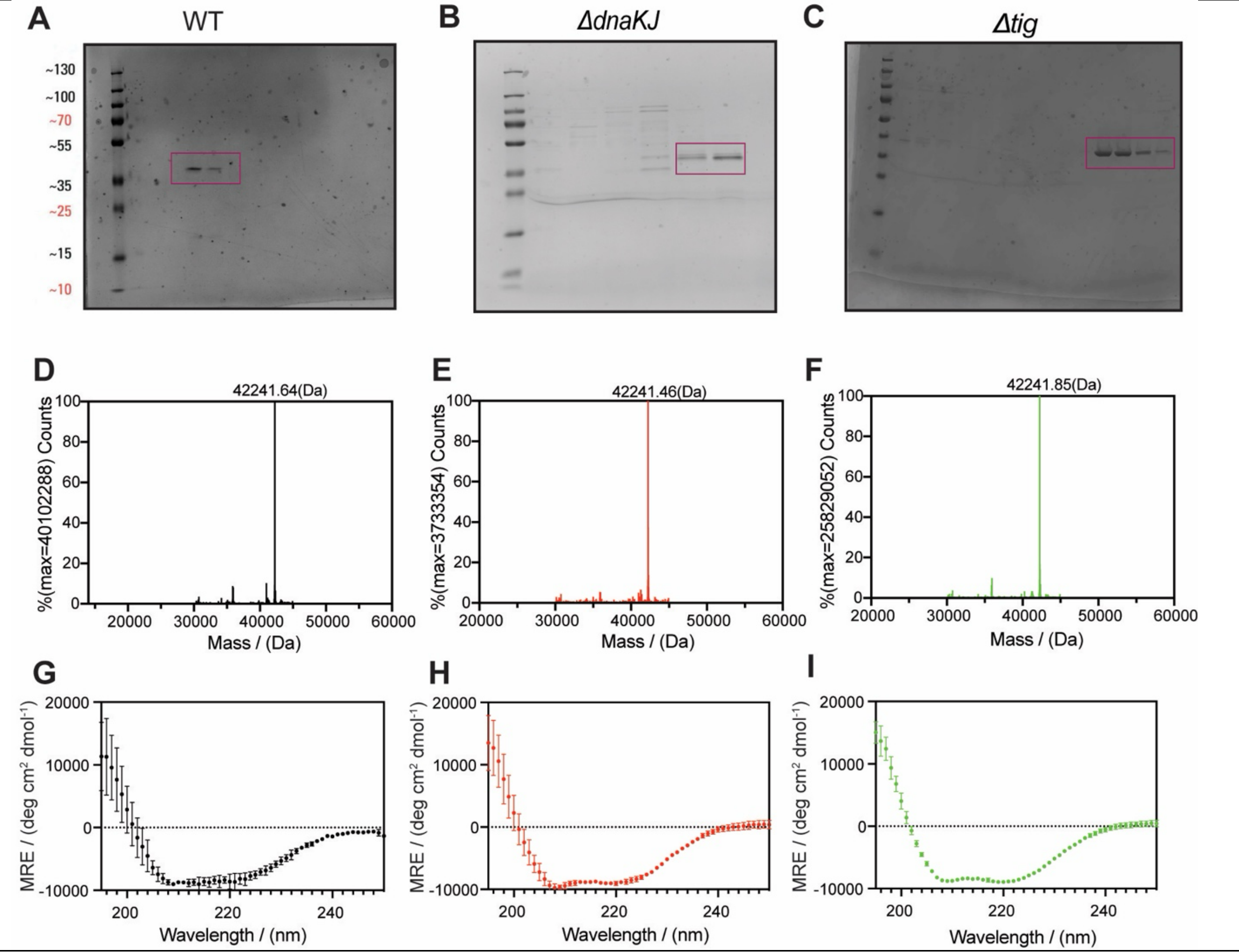

Appendix Figure S3. Biochemical and biophysical validation and structural characterization of genomically tagged PGK-StrepTagII purified from WT and chaperone knockout *E. coli* strains. (A-C) SDS-PAGE analysis (10% TG-SDS) of PGK-StrepTagII purified via anion exchange from WT (A), Δ*dnaKJ* (B), and Δ*tig* (C) *E. coli* strains. Coomassie staining reveals a dominant band corresponding to the expected molecular weight (~42 kDa), indicating high purity across all backgrounds. (D-F) Intact mass spectrometry of purified PGK from WT (D), Δ*dnaKJ* (E), and Δ*tig* (F) confirms precise mass of ~42,241 Da in all cases, supporting correct C-terminal tagging and the absence of truncations or modifications. (G-I) Far-UV circular dichroism (CD) spectra of PGK-Strep purified from WT (G), Δ*dnaKJ* (H), and Δ*tig* (I) strains. Points correspond to means and error bars correspond to standard deviations of ellipticity values obtained from purified protein samples from at least three different preparations.

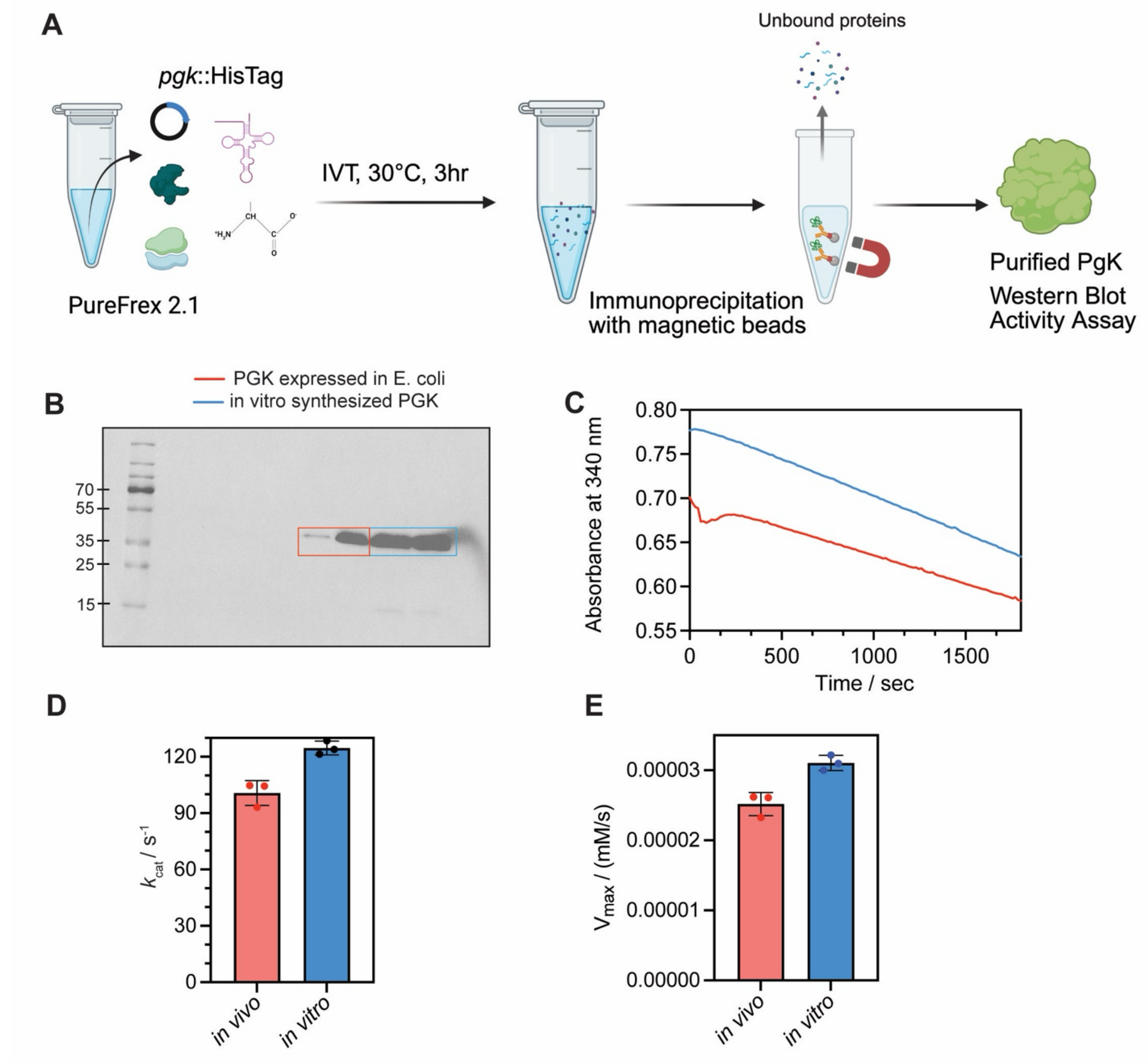

Appendix Figure S4. Functional validation of PGK synthesized in vitro using the PureFrex 2.1 system. (A) Schematic of in vitro translation (IVT) and purification of His-tagged PGK using the PUREfrex 2.1 system. The *pgk::HisTag* plasmid was added to the IVT reaction mixture and incubated at 30°C for 3 h. PGK was subsequently purified using Ni-NTA magnetic beads. (B) Western blot confirming successful synthesis of PGK. Lane 1-2: PGK expressed from *E. coli.* Lane 3-4: PGK synthesized *in vitro* using PUREfrex. Both bands migrate at the expected size. (C) Sample raw time-course data of PGK activity monitored by NADH absorbance decrease at 340 nm using a coupled assay (3-phosphoglycerate [3-PG] = 5 mM, [PGK] = 0.00025 µM for both). (D, E) Saturating rate constant of PGK produced *in vivo* (red) and *in vitro* (blue). Bar graphs show k_{cat} (D) and V_{max} (E), demonstrating that PGK synthesized *in vitro* is enzymatically active and comparable in activity to its *in vivo*-expressed counterpart. Points represent values for three technical replicates and error bars represent standard deviation. See Source Data for Figure S4D-E.

Appendix Table S1. Primer sequences used in this study for scarless CRISPR-mediated Strep-TagII knock-in at the *pgk* locus.

Primer Name	Sequence
ColonyPCR_*pgK*_F_knockIN	5' aaccattctgtggaacgg
ColonyPCR_*pgK*_R	5' tttcgaattgtggatgactcc
Amplify_geneblock_*pgK*_F	5' caaaatctcctacatctccactggcgg
Amplify_geneblock_*pgK*_R	5' tgtcgccttcctgcaactcgaattatttagag
Backbone_Cas9_F	5' tttgatatcgagctcgc
Backbone_Cas9_R	5' tttagcttccttagctcctg
QuickChange_pkD_*pgK*_f_protospacer	5' gctaagaagtaaaaaatcacgtgctcagtatctctatcactga
QuickChange_pkd_*pgK*_r_protospacer	5' gtgatttttacttcttagcgttttagagctagaaatagcaag
