# Supplementary material for: Chaperone dependency during biogenesis does not correlate with chaperone dependency during refolding
Source: Mol Syst Biol. 2025 Oct 28;22(1):139–64. doi: 10.1038/s44320-025-00166-6 (PMC12759075; doi:10.1038/s44320-025-00166-6)
Supplement: Supplementary file 9 — Expanded View Figures [file 44320_2025_166_MOESM9_ESM.pdf]

Expanded View Figures

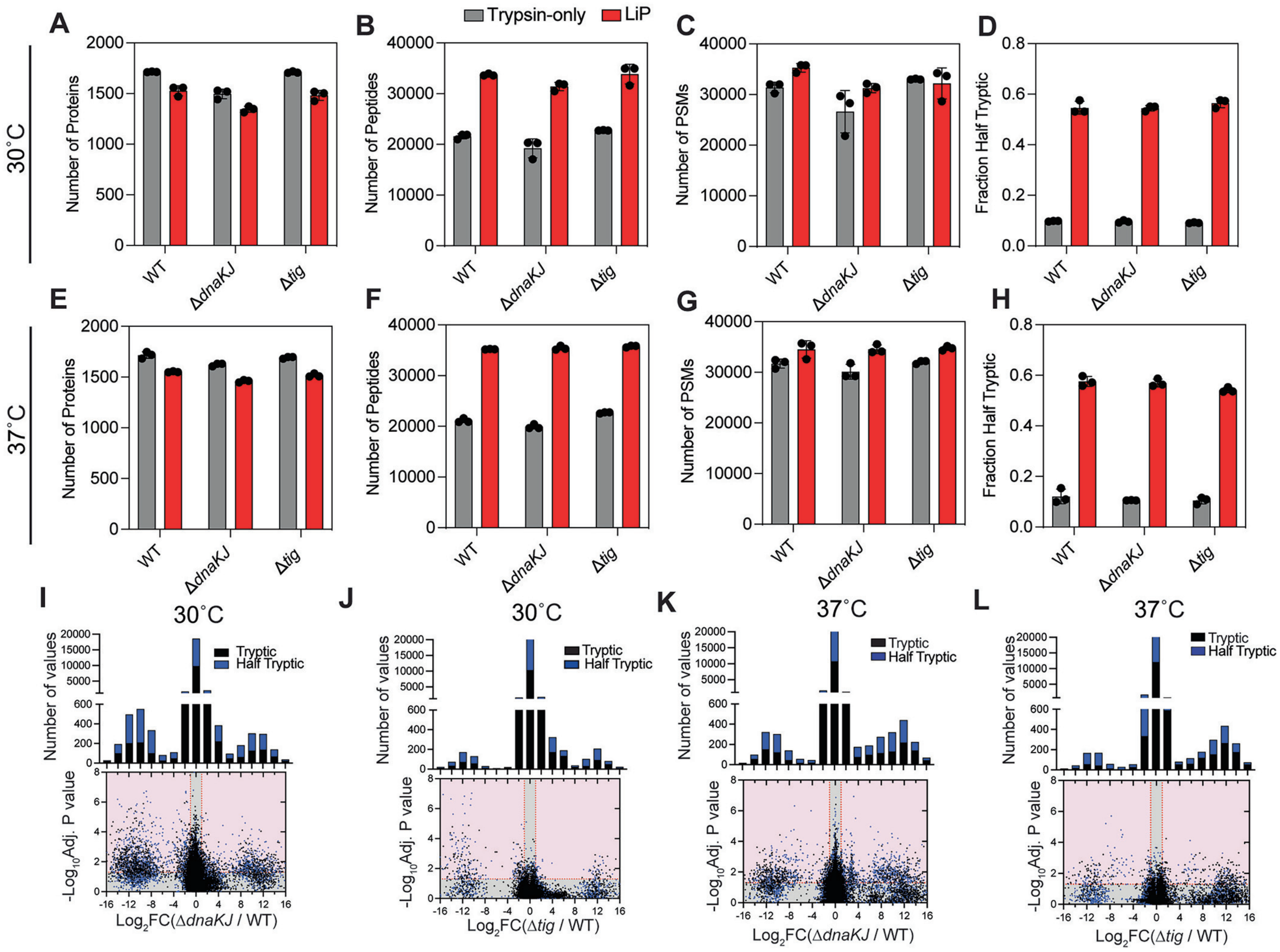

Figure EV1. Quality control metrics and peptide type distributions in LiP-MS analysis across chaperone knockout strains.

Number of proteins (**A**, **E**), peptides (**B**, **F**), and peptide-spectrum matches (PSMs) (**C**, **G**) identified per sample injection/bio-replicate from WT, Δ*dnaKJ*, and Δ*tig* strains at 30 °C (top row) and 37 °C (bottom row) ($n = 3$). LiP samples (red bars) were subjected to limited proteolysis using Proteinase K followed by trypsin digestion, whereas trypsin-only controls (gray bars) underwent only tryptic digestion. Each dot represents a biological replicate ($n = 3$). Bars represent means and error bars represent standard deviation. (**D**, **H**) Fraction of peptides that are half-tryptic (i.e., one non-tryptic terminus attributed to Proteinase K cleavage) in each sample. (**I**–**L**) Volcano plots showing changes in cut-sites abundance (half-tryptic in blue; tryptic in black) between WT and chaperone knockout strains: (**I**) Δ*dnaKJ*/WT at 30 °C, (**J**) Δ*tig*/WT at 30 °C, (K) Δ*dnaKJ*/WT at 37 °C, (**L**) Δ*tig*/WT at 37 °C. Peptides with >2-fold change and $P < 0.05$ (Welch's *t* test following protein-wise Benjamini–Hochberg FDR correction) are highlighted in pink-shaded regions. Insets above each volcano plot show the distribution of tryptic and half-tryptic peptides across $\log_2$ fold-change bins. All peptide quantifications are available in the associated Zenodo deposition (10.5281/zenodo.16879364).

 © The Author(s)

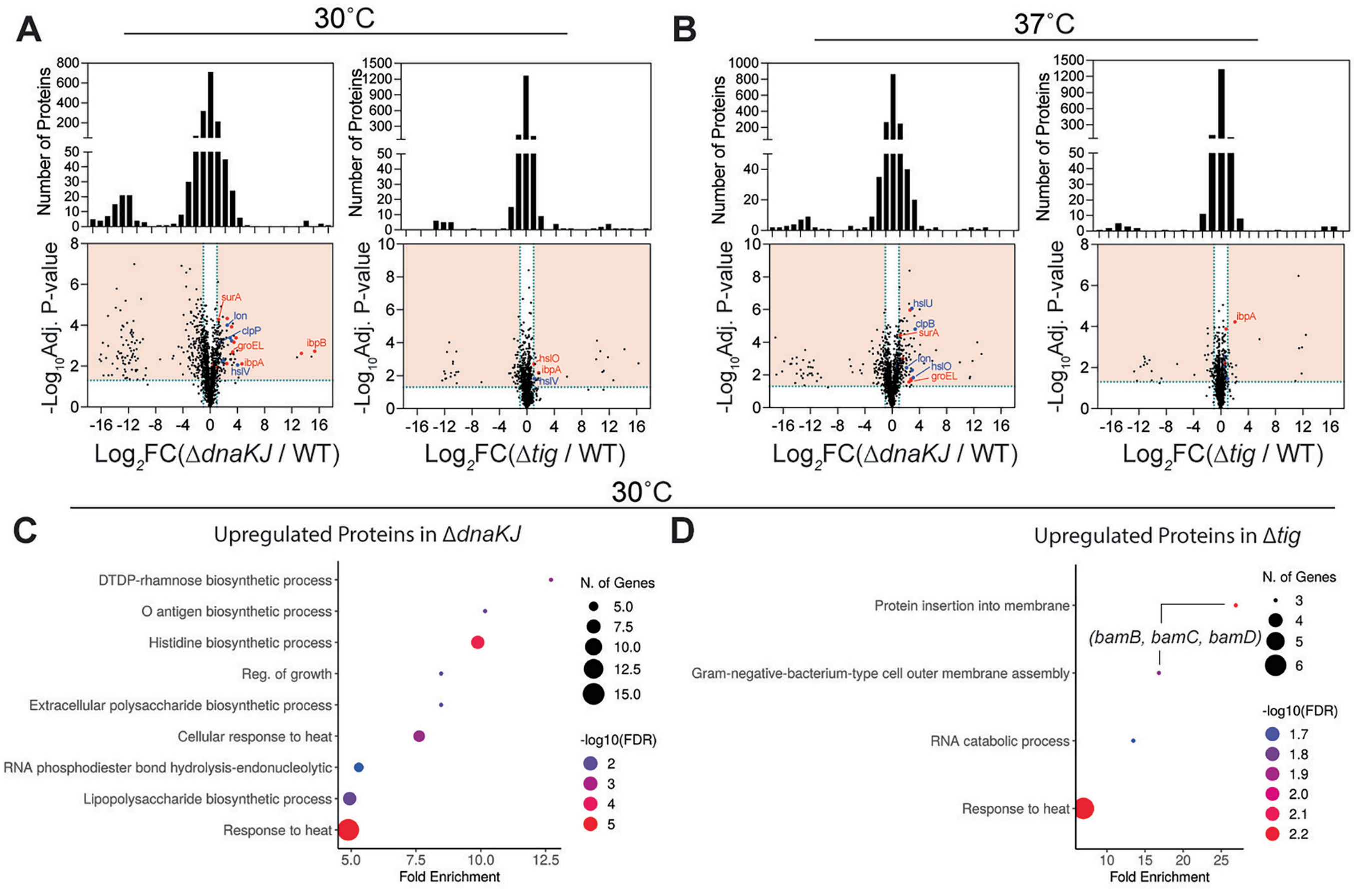

Figure EV2. Global Protein abundance changes and GO enrichment analyses in Δ*dnaKJ* and Δ*tig* strains at 30 °C and 37 °C.

(**A**, **B**) Volcano plots showing differential protein abundance in Δ*dnaKJ* (left panels) and Δ*tig* (right panels) relative to WT at 30 °C (**A**) and 37 °C (**B**). Each dot represents an individual protein. Significantly altered proteins (log_2 fold change > 1 or < –1; adjusted $P < 0.05$; as calculated from Fragpipe) are highlighted in the shaded regions. Histograms above each plot display the distribution of fold changes. Molecular chaperones are labeled in red, and proteases in blue (see Source Data for Fig. EV2A,B). (**C**, **D**) Gene Ontology (GO) Biological Process enrichment for proteins significantly upregulated in Δ*dnaKJ* (**C**) and Δ*tig* (**D**) at 30 °C. Dot size represents the number of genes enriched in each term; color indicates FDR-adjusted significance, as calculated by ShinyGo v.0.80. Δ*dnaKJ* cells show enriched processes related to heat response, biosynthesis, and polysaccharide metabolism, while Δ*tig* upregulated proteins are enriched in membrane insertion, cell envelope assembly, and RNA catabolism. Source data are available online for this figure.

© The Author(s)

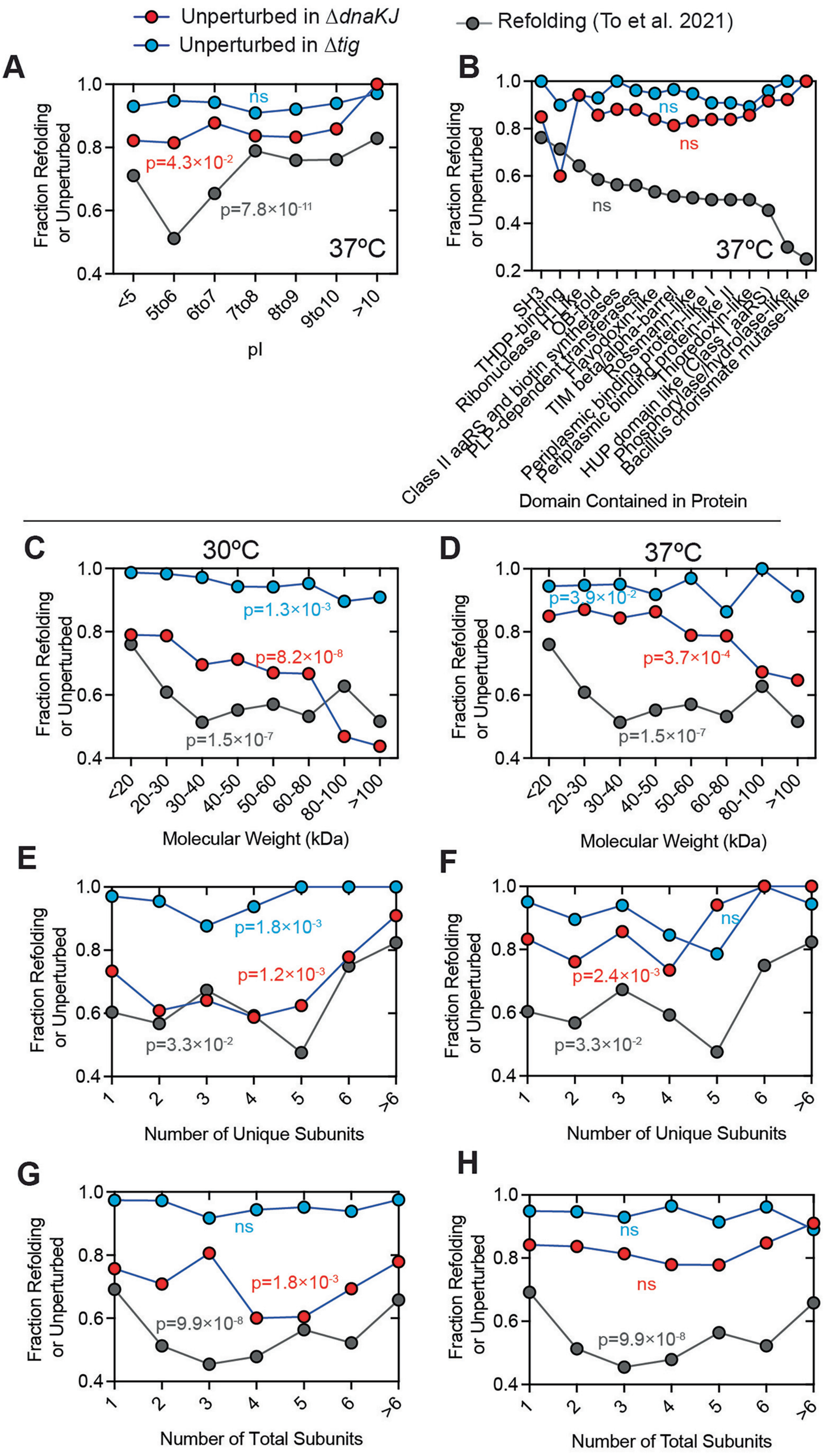
Unperturbed in ΔdnaKJ
Unperturbed in Δtig
Refolding (To et al. 2021)
A
Fraction Refolding or Unperturbed
ns
p=4.3×10-2
p=7.8×10-11
37ºC
<5
5to6
6to7
7to8
8to9
9to10
>10
pI
B
ns
ns
ns
37ºC
SH3
THDP-binding
Ribonuclease H-like
OB-fold
Class II aaRS and biotin synthetases
PLP-dependent transferases
Flavodoxin-like
TIM beta/alpha-barrel
Rossmann-like
Ring protein-like I
Periplasmic binding protein-like II
Periplasmic binding protein-like I
Thioredoxin-like
HUP domain like (Class I aaRS)
Phosphorylase/hydrolase-like
Bacillus chorismate mutase-like
Domain Contained in Protein
C
30ºC
p=1.3×10-3
p=8.2×10-8
p=1.5×10-7
<20
20-30
30-40
40-50
50-60
60-80
80-100
>100
Molecular Weight (kDa)
D
37ºC
p=3.9×10-2
p=3.7×10-4
p=1.5×10-7
Molecular Weight (kDa)
E
p=1.8×10-3
p=1.2×10-3
p=3.3×10-2
Number of Unique Subunits
F
ns
p=2.4×10-3
p=3.3×10-2
Number of Unique Subunits
G
ns
p=1.8×10-3
p=9.9×10-8
Number of Total Subunits
H
ns
ns
p=9.9×10-8
Number of Total Subunits

 © The Author(s)

Figure EV3. Comparison of chaperone dependency during primary folding and in vitro refolding.

Fraction of proteins that refold in vitro following chemical denaturation (gray; data from To et al, 2021) versus those that remain structurally unperturbed in vivo in either Δ*dnaKJ* (red) or Δ*tig* (blue) backgrounds, measured using LiP-MS at 30 °C (left panels) and 37 °C (right panels). Proteins are grouped by various properties: (**C**, **D**) Molecular weight (kDa); (**E**, **F**) Number of unique subunits; (**G**, **H**) Total number of subunits; (**A**) Isoelectric point (pI) at 37 °C; (**B**) Fold-type domain annotations according to ECOD X-group, at 37 °C. *P* values calculated with the chi-square test (see Source Data for Fig. EV3). Source data are available online for this figure.

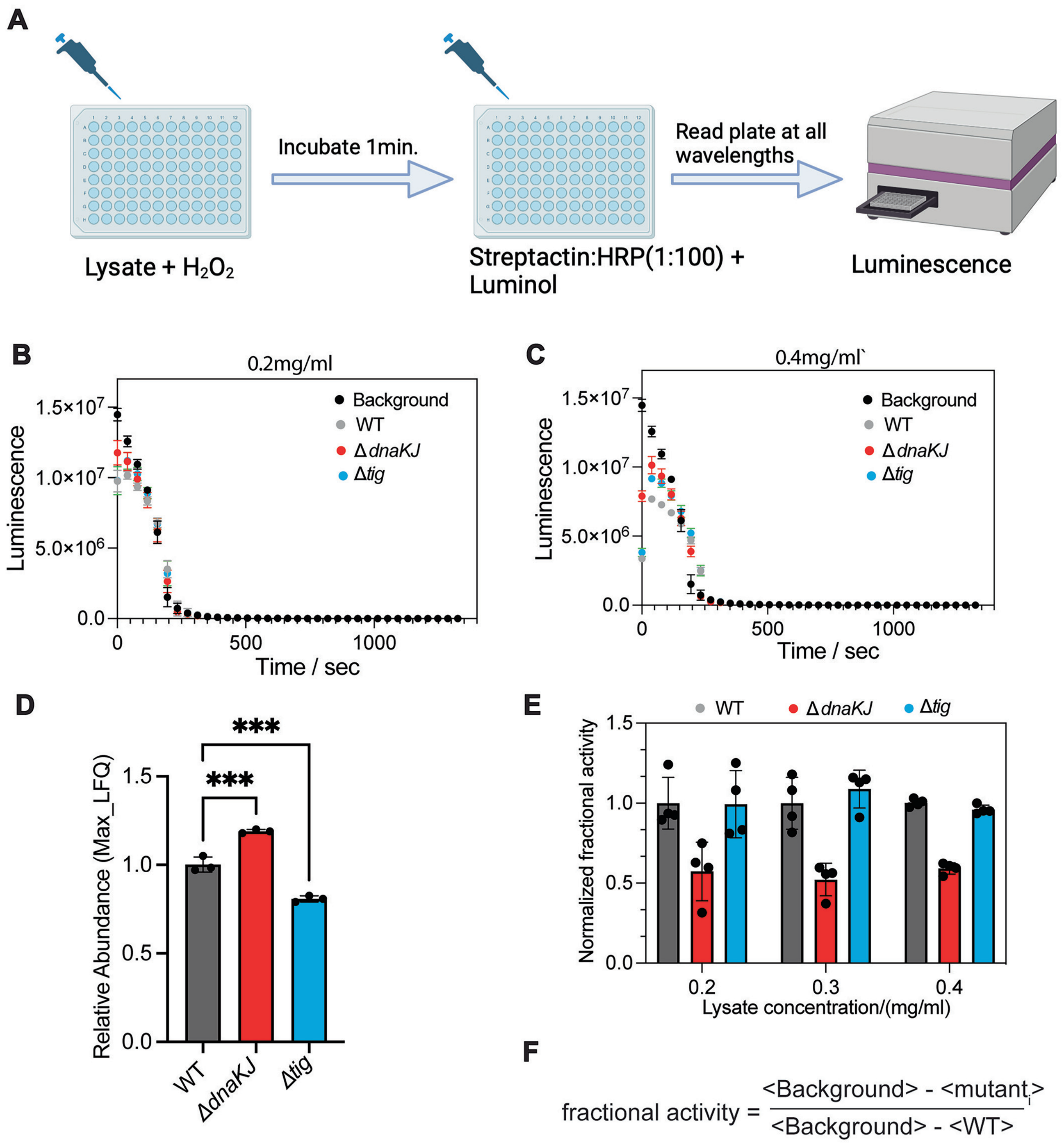

$$\text{fractional activity} = \frac{\langle\text{Background}\rangle - \langle\text{mutant}_i\rangle}{\langle\text{Background}\rangle - \langle\text{WT}\rangle}$$

 © The Author(s)

Figure EV4. Luminescence-based catalase activity assay confirms functional impairment of KatG in Δ*dnaKJ* background.

(**A**) Schematic of the assay workflow. Clarified lysates were incubated with H_2O_2 for 1 min, after which residual H_2O_2 was detected by adding streptavidin-HRP and luminol, with luminescence measured in real-time. (**B**, **C**) Raw luminescence decay curves over time for KatG activity in WT, Δ*dnaKJ*, and Δ*tig* lysates at two concentrations for 4 biological replicates ($n = 4$): 0.2 mg/mL (**B**) and 0.4 mg/mL (**C**). Background control lacking lysate is shown in black. Points represent means and error bars represent standard deviation (see Source Data for Fig. EV4B,C). (**D**) Relative abundance of KatG across strains as measured by MaxLFQ protein quantification ($n = 3$). P values were calculated using ordinary one-way ANOVA followed by Tukey's multiple comparisons test ($\alpha = 0.05$). Asterisks indicate statistical significance as follows: $^{***}P < 0.001$ (see Source Data for Fig. EV4D). (**E**) Normalized fractional catalase activity at varying lysate concentrations. Activity in Δ*dnaKJ* is consistently reduced across all concentrations, whereas Δ*tig* retains near-WT activity. Bars represent means and error bars represent standard deviation (see Source Data for Fig. EV4E). (**F**) Formula used to calculate fractional activity relative to WT and background controls. Source data are available online for this figure.

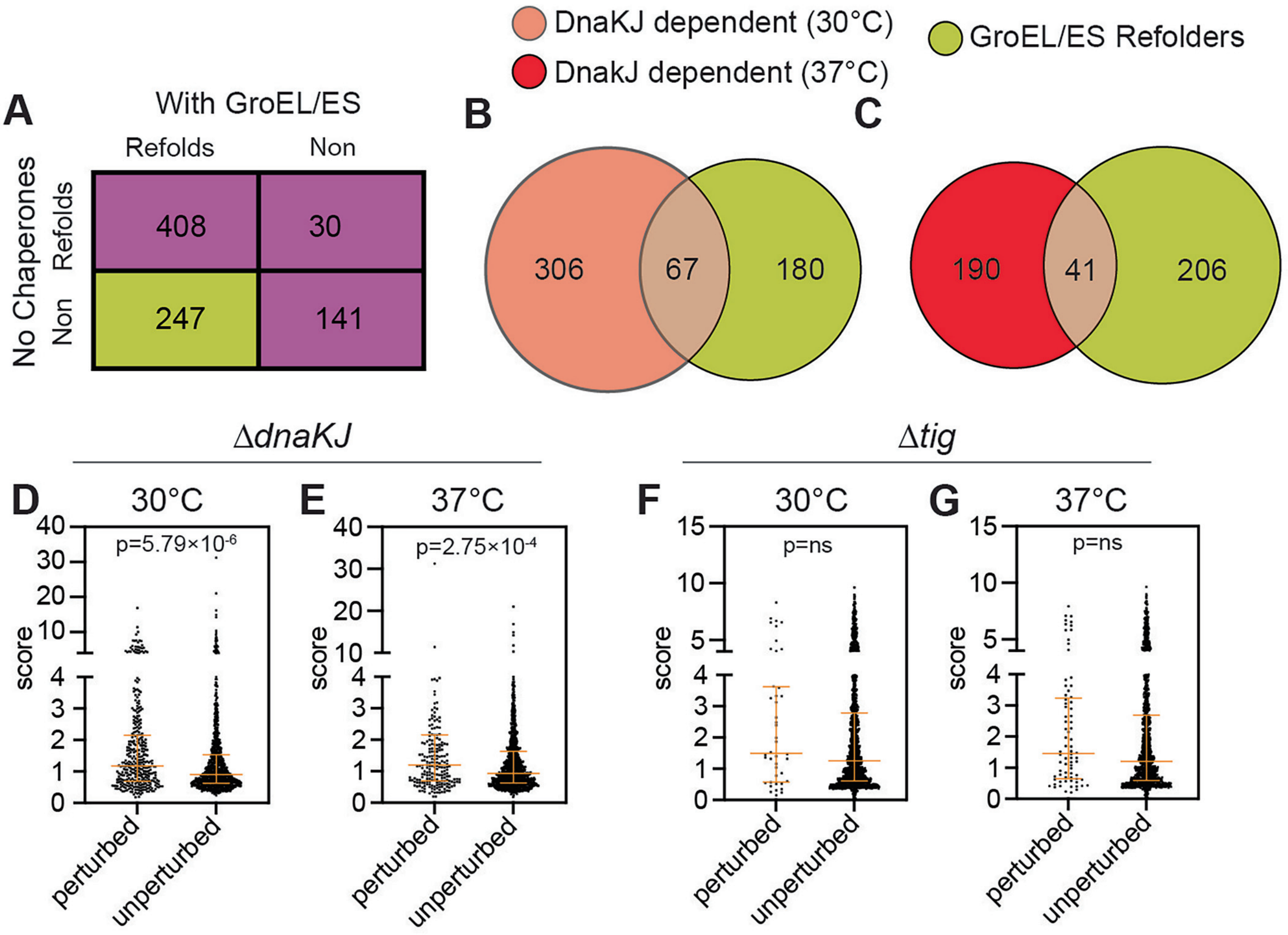

Figure EV5. Comparison of GroEL/ES-assisted refolding with DnaKJ dependence at different temperatures and comparison of in vivo Δ*dnaKJ* perturbation data with chaperone binding scores from Galmozzi et al, 2025.

(**A**) Contingency table from chaperone refolding experiments (To et al, 2022) that defines which proteins are categorized as GroEL-dependent refolders (see Source Data for Fig. EV5A). (**B**) Overlap between in vitro GroEL-dependent refolders ($n = 247$) and in vivo DnaKJ-dependent substrates ($n = 373$) identified by structural perturbation in Δ*dnaKJ* cells at 30 °C. (**C**) Same comparison as in (**B**), but using in vivo Δ*dnaKJ* LiP-MS data at 37 °C ($n = 231$) (see Source Data for Fig. EV5B,C). (**D–G**) Scatter plots show chaperone binding scores (y-axis), reflecting binding affinity to the DnaKJ chaperone from Galmozzi et al, 2025, for proteins classified as perturbed ("p") or unperturbed ("u") in our in vivo LiP-MS dataset. (**D**) Δ*dnaKJ* strain at 30 °C ($n_p = 373$, $n_u = 974$). (E) Δ*dnaKJ* strain at 37 °C ($n_p = 231$, $n_u = 1120$). (**F**) Δ*tig* strains at 30 °C ($n_p = 45$, $n_u = 1298$). (**G**) Δ*tig* strains at 37 °C ($n_p = 81$, $n_u = 1271$). *P* values (shown) were calculated using the non-parametric Kolmogorov–Smirnov test. Horizontal orange lines represent median ± interquartile range (see Source Data for Fig. EV5D,E and EV5F,G). Source data are available online for this figure.

 © The Author(s)
